# Adeno‐associated virus serotype 1‐based gene therapy for FTD caused by *GRN* mutations

**DOI:** 10.1002/acn3.51165

**Published:** 2020-09-16

**Authors:** Christian Hinderer, Rod Miller, Cecilia Dyer, Julia Johansson, Peter Bell, Elizabeth Buza, James M. Wilson

**Affiliations:** ^1^ Gene Therapy Program Perelman School of Medicine University of Pennsylvania Philadelphia Pennsylvania USA

## Abstract

**Objective:**

Dominant loss‐of‐function mutations in the gene encoding the lysosomal protein, progranulin, cause 5‐10% of frontotemporal dementia cases. As progranulin undergoes secretion and endocytosis, a small number of progranulin‐expressing cells can potentially supply the protein to the entire central nervous system. Thus, gene therapy is a promising treatment approach.

**Methods:**

We evaluated adeno‐associated viral vector administration into the cerebrospinal fluid as a minimally invasive approach to deliver the granulin gene to the central nervous system in a murine disease model and nonhuman primates.

**Results:**

In progranulin‐deficient mice, vector delivery into the lateral cerebral ventricles increased progranulin levels in the cerebrospinal fluid and normalized histological and biochemical markers of progranulin deficiency. A single vector injection into the cisterna magna of nonhuman primates achieved CSF progranulin concentrations up to 40‐fold higher than those of normal human subjects and exceeded CSF progranulin levels of successfully treated mice. Animals treated with an adeno‐associated virus serotype 1 vector exhibited progranulin expression fivefold higher than those treated with an AAV5 vector or the AAV9 variant, AAVhu68, apparently due to remarkably efficient transduction of ependymal cells. Progranulin expression mediated by adeno‐associated viral vectors was well tolerated in nonhuman primates with no evidence of dose‐limiting toxicity, even at vector doses that induced supraphysiologic progranulin expression.

**Interpretation:**

These findings support the development of AAV1‐based gene therapy for frontotemporal dementia caused by progranulin deficiency.

## Introduction

Up to 10% of all cases of frontotemporal dementia (FTD) are caused by loss‐of‐function mutations in the granulin (*GRN*) gene, which encodes the lysosomal protein progranulin (PGRN).^1^ *GRN* mutations are inherited in an autosomal dominant fashion with greater than 90% penetrance by age 70.^1^ GRN mutation carriers typically present with clinical phenotypes of behavioral variant FTD or primary progressive aphasia in the fifth or sixth decade of life, and universally exhibit a progressive course, with average survival of 6years from symptom onset.^2^ There are currently no disease‐modifying therapies for FTD caused by *GRN* mutations (FTD‐GRN).

Several lines of evidence suggest that the pathophysiology of FTD‐GRN is related to lysosomal dysfunction secondary to decreased progranulin levels. While inheritance of a single *GRN* mutation causes FTD, patients with homozygous loss‐of‐function mutations present much earlier in life with neuronal ceroid lipofuscinosis (NCL, Batten disease), which is characterized by the accumulation of auto‐fluorescent material (lipofuscin) in the lysosomes of neurons, rapid cognitive decline, and retinal degeneration.^3^ Although FTD patients with a single *GRN* mutation experience later symptom onset, they ultimately develop lysosomal storage lesions in the brain and retina identical to those of NCL patients prior to the first clinical manifestations of the disease.^4^ Recent studies show that PGRN plays a critical role in lysosomal function by promoting lysosome acidification and serving as a chaperone for lysosomal proteases, including cathepsin D (CTSD).^5^, ^6^ Mutations in the gene that encodes CTSD can also lead to an NCL phenotype, which supports a common pathophysiology related to deficient lysosomal protease activity.^7^

Like many lysosomal proteins, newly synthesized PGRN can either be transported directly from the trans‐Golgi network to the lysosome or secreted. PGRN that is released into the extracellular fluid can be endocytosed and transported to the lysosomes of other cells.^6^ This uptake is mediated by direct binding of PGRN to sortilin on the cell surface, or via PGRN binding to prosaposin, a lysosomal protein that is taken up via mannose‐6‐phosphate receptors.^8^ An important consequence of the secretion and endocytosis of PGRN was demonstrated in transgenic mice that lack *GRN* selectively in neurons.^9^ In contrast to mice with global *GRN* deletion, ablating PGRN expression in neurons alone does not cause neuronal lipofuscin accumulation, which indicates that other cells can supply the protein to neurons.^9^ Overexpressing PGRN in a subset of cells may therefore be sufficient for widespread delivery of the protein to the brain, making gene therapy a potentially effective approach even with modest gene‐transfer efficiency.

A prior study demonstrated that adeno‐associated viral (AAV) vector delivery to the brain parenchyma of *GRN*‐knockout mice corrected lysosomal storage lesions, particularly in the regions surrounding the injection site.^10^ Translating an AAV‐based gene therapy approach to patients with FTD‐GRN will require identification of a vector capsid and method of administration that can safely achieve widespread PGRN delivery in the brain. Using a nonhuman primate model, we evaluated a minimally invasive intra‐cisterna magna (ICM) approach to deliver PGRN‐expressing AAV vectors into the cerebrospinal fluid.^11^, ^12^, ^13^ In addition, we compared PGRN expression in NHPs treated with diverse AAV capsid serotypes. Our findings highlight the critical role of capsid serotype in achieving high levels of PGRN expression following ICM administration, and demonstrate the safety and feasibility of this approach in NHPs, paving the way for human trials.

## Materials and Methods

### Animal procedures

We performed animal studies at the University of Pennsylvania, an AAALAC‐accredited facility. The Institutional Animal Care and Use Committee of the University of Pennsylvania approved all animal protocols. We purchased breeding pairs of *GRN*‐knockout mice from The Jackson Laboratory (stock #0131730) and maintained a colony at the University of Pennsylvania. Wild‐type C57BL/6 mice (stock #000664) served as controls. In the first study, we anesthetized two‐month‐old mice (n = 10 per group) with isoflurane and injected 1 × 10^11^ vector genome copies (GC) of AAVhu68 expressing human PGRN or vehicle in a volume of 5 µL into the lateral cerebral ventricle (ICV). Sixty days post‐injection, mice received ketamine/xylazine anesthesia and were euthanized by exsanguination; we confirmed death by cervical dislocation. In the second study, *GRN*‐knockout mice (n = 8 per group) were treated at seven months of age and sacrificed at 11 months of age. At the time of necropsy, we collected blood by cardiac puncture into serum‐separator tubes; we collected CSF via suboccipital puncture using a 32‐gauge needle connected to polyethylene tubing. Serum and CSF samples were frozen on dry ice and stored at −80°C until analysis. We collected the frontal cortex for biochemistry and immediately froze the tissue on dry ice; the rest of the brain was fixed in 10% formalin for histology.

We purchased 3‐ to 4‐year‐old rhesus macaques from Envigo. Six animals were included in the GFP vector study (n = 2 per vector) and six animals were included in the PGRN vector study (n = 2 per vector). For intrathecal vector administration, we sedated animals with an intramuscular injection of dexmedetomidine and ketamine and administered a single ICM injection of 3 × 10^13^ GC of each AAV vector in 1 mL of artificial CSF. We verified needle placement via myelography using a fluoroscope (OEC9800 C‐Arm, GE), as previously described.^14^ We administered the analgesic meloxicam to all animals post‐injection. We collected CSF prior to injection and thereafter on a weekly basis via suboccipital puncture. Automated CSF cell counts with manual differential was performed by Antech Diagnostics. Animals were euthanized by barbiturate overdose. The collected tissues were immediately frozen on dry ice or fixed in 10% formalin for histology.

### Vector production

Vector production is described in supplemental methods.

### Statistics

We performed comparisons of hexosaminidase enzymatic activity, lipofuscin counts, and CD68+ area in wild‐type, *GRN*‐knockout and AAV‐treated *GRN*‐knockout mice using a one‐way analysis of variance followed by a post‐hoc Tukey’s multiple comparisons test.

## Results

### Proof‐of‐concept studies in a murine disease model

We utilized a murine disease model to assess AAV‐based gene therapy for PGRN deficiency. Mice heterozygous for *GRN* mutations (*GRN^+/−^*) have been reported to exhibit subtle behavioral abnormalities but not the pathological hallmarks of *GRN*‐related neurodegenerative disease, likely because the mouse lifespan does not allow for the development of the sequelae of *GRN* haploinsufficiency, which manifest after several decades in humans.^15^, ^16^, ^17^, ^18^ In contrast, complete PGRN deficiency in *GRN^−/−^* mice recapitulates early hallmarks of *GRN* haploinsufficiency in humans, such as impaired lysosomal function, accumulation of auto‐fluorescent lysosomal storage material (lipofuscin), and activation of microglia.^16^, ^19^ However, *GRN^−/−^* mice do not exhibit neuronal loss even up to two years of age.^16^, ^19^ Despite the absence of overt neurodegeneration or neurological signs, the biochemical and histological similarities to *GRN* haploinsufficiency in humans make *GRN^−/−^* mice a potentially informative model to evaluate novel therapies.

To aid in the design of pharmacology studies, we first evaluated the natural history of brain pathology in the *GRN^−/−^* mouse model. Progressive accumulation of lipofuscin deposits was apparent in the cortex, hippocampus, and thalamus (Fig. S1). Lipofuscin was apparent as early as 2 months of age, consistent with previous findings.^20^ To evaluate lysosomal function, we measured activity of the lysosomal enzyme, hexosaminidase, which is upregulated in the setting of lysosomal storage.^15^, ^21^, ^22^ In contrast to the progressive accumulation of lipofuscin, brain hexosaminidase activity was similarly elevated in *GRN^−/−^* mice of all ages (Fig. S1).

We performed our initial studies with an AAV vector based on the natural isolate, AAVhu68, which is closely related to the clade F isolate AAV9. We treated *GRN^−/−^* mice at two to three months of age with an intracerebroventicular (ICV) injection of either an AAVhu68 vector expressing human *GRN* or vehicle (phosphate‐buffered saline/PBS; N = 10 per group). In addition, we injected a cohort of wild‐type mice with vehicle (N = 10). Previous studies demonstrated that ICV administration of AAV vectors at similar doses results in transduction limited to brain regions near the injected ventricle.^23^ Thus, this is a useful system to evaluate whether global improvements in brain lesions can be achieved through the secretion of PGRN by a small population of cells.

**Figure 1:**
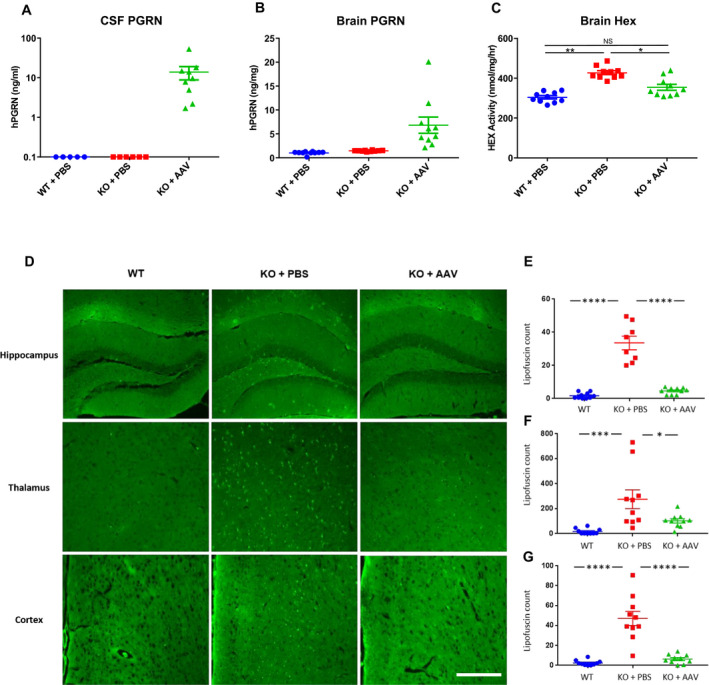
AAV‐mediated PGRN expression corrects lysosomal pathology in brains of young adult GRN^−/−^ mice. GRN^−/−^ mice (KO) or GRN^+/+^ (WT) controls were treated with a single ICV injection of vehicle (PBS) or an AAVhu68 vector expressing human PGRN (10^11^ GC) at two months of age (N = 10 per group). Animals were sacrificed 60 days after injection, and human PGRN was measured in (A) CSF and (B) the frontal lobes of the brain by ELISA. C, We measured hexosaminidase activity in brain samples. Brain PGRN concentration and Hex activity were normalized to total protein. D, We imaged unstained brain sections for auto‐fluorescent material (lipofuscin) in hippocampus, thalamus, and frontal cortex. E‐G, A blinded reviewer quantified lipofuscin deposits in hippocampus, thalamus, and frontal cortex. Lipofuscin counts are expressed per high‐power field. N = 10 per group except KO + PBS hippocampus, which is N = 8. *P < 0.05, **P < 0.005, ***P < 0.001, ****P < 0.0001, one‐way ANOVA followed by Tukey’s multiple comparisons test. Scale bar = 250 µm (cortex and thalamus), 500 µm (hippocampus).

Two months after vector administration, we euthanized the animals and collected brain, CSF, and serum. We were able to detect human PGRN in the brains and CSF of AAV‐treated *GRN^−/−^* mice (Fig. 1). Expression of PGRN was accompanied by normalization of lysosomal enzyme expression, with hexosaminidase activity returning to near‐normal levels in the brains of AAV‐treated *GRN^−/−^* mice (Fig. 1). Compared to vehicle‐treated *GRN* ^−/−^ mice, AAV‐treated *GRN* ^−/−^ mice exhibited reduced lipofuscin deposits in all examined brain regions (Fig. 1).

**Figure 2:**
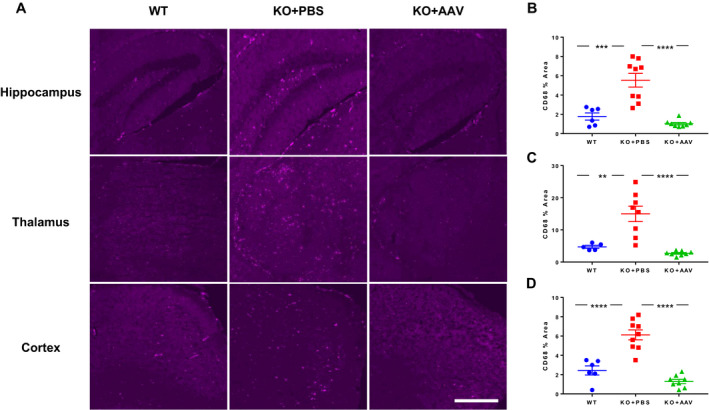
AAV‐mediated PGRN expression corrects brain microgliosis in aged GRN^−/−^ mice. GRN^−/−^ mice (KO) or GRN^+/+^ (WT) controls were treated with a single ICV injection of vehicle (PBS) or an AAVhu68 vector expressing human PGRN (10^11^ GCs) at seven months of age. A, Animals were sacrificed four months after injection, and brain sections were stained for CD68. B‐D, A blinded reviewer quantified CD68‐positive areas in images of hippocampus, thalamus, and frontal cortex using ImageJ software. Areas are expressed per high‐power field. **P < 0.005, ***P < 0.001, ****P < 0.0001, one‐way ANOVA followed by Tukey’s multiple comparisons test. Scale bar = 500 µm.

The initial proof‐of‐concept study demonstrated the therapeutic activity of AAV‐mediated PGRN expression in mice treated at an early age, when storage material has just begun to appear in the brain. We subsequently evaluated the impact of gene transfer in older mice with more severe pre‐existing pathology. We administered a single ICV injection of an AAVhu68 vector expressing human PGRN or vehicle to seven‐month‐old *GRN^−/−^* mice. At 11 months of age – in addition to extensive brain lipofuscin deposits (Fig. S2) – *GRN^−/−^* mice exhibited marked microgliosis, similar to patients with FTD caused by *GRN* mutations (Fig. 2).^24^ *GRN* gene transfer reduced brain hexosaminidase activity and lipofuscin deposits in aged mice similar to our findings in younger animals (Fig. S2). In addition, microgliosis was completely corrected in the brains of treated mice (Fig. 2).

### Evaluating AAV‐mediated GRN gene transfer in NHPs

**Figure 3:**
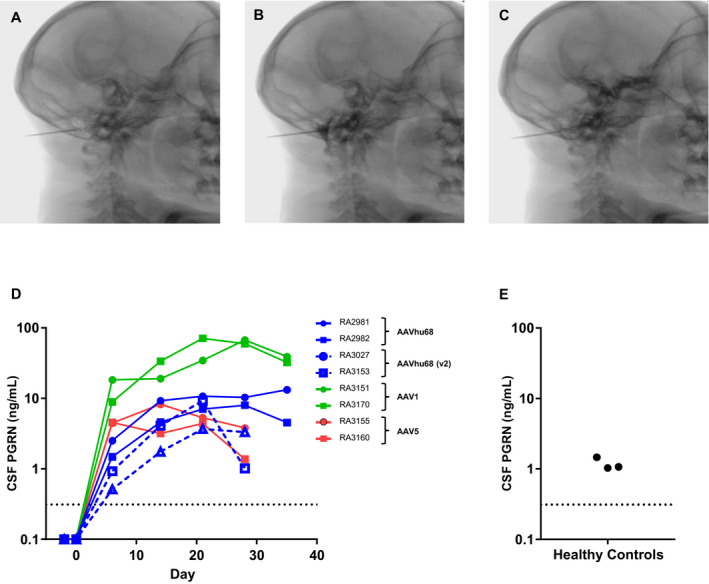
Human PGRN expression in the CSF of rhesus macaques following ICM AAV delivery. Adult rhesus macaques were administered AAV1, AAV5, or AAVhu68 vectors (3 × 10^13^ GC) expressing human PGRN from a chicken beta‐actin promoter by ICM injection on study day 0 (N = 2 per vector). Two additional macaques were administered an AAVhu68 vector expressing hPGRN from a ubiquitin C promoter (AAVhu68 V2). We performed the ICM injection under fluoroscopic guidance. A, After confirming needle placement by fluoroscopy and CSF return, (B) injection of contrast material demonstrated distribution within the cisterna magna. C, Displacement of the contrast was apparent during subsequent vector infusion. We measured human PGRN in CSF of (D) treated macaques and (E) healthy adult human subjects by ELISA. Dotted line = limit of quantification for hPGRN in CSF at a 1:5 dilution.

Our findings in *GRN^−/−^* mice demonstrate that delivering an AAV vector into CSF can 1) achieve sufficient brain transduction to produce therapeutic levels of PGRN; and 2) prevent or reverse biochemical and histological findings associated with PGRN deficiency. In order to translate this approach to humans, we carried out studies in NHPs using a clinically relevant route of vector administration. ICM AAV delivery into the CSF is a minimally invasive approach that results in more extensive brain transduction than administration via lumbar puncture.^13^ In order to identify a vector capable of achieving optimal expression levels of human PGRN in CSF, we tested AAV serotypes 1, 5 and hu68, which represent distinct clades of natural AAV isolates. These vectors carried a human *GRN* transgene driven by a chicken beta‐actin promoter with a cytomegalovirus immediate‐early enhancer. We also tested an AAVhu68 vector carrying a human ubiquitin C promoter. We detected robust PGRN expression in the CSF of all NHPs following vector administration (Fig. 3). The CSF of the two animals treated with the AAVhu68 vector exhibited human PGRN levels that were up to tenfold greater than levels of healthy human controls. These PGRN levels were similar to those that reversed lysosomal abnormalities in the brains of *GRN* ^−/−^ mice. The CSF expression levels were roughly equivalent for both the AAV5‐ and the AAVhu68‐treated groups. Expression of human PGRN was greatest in the animals treated with an AAV1 vector, reaching more than 40‐fold normal human levels.

**Figure 4:**
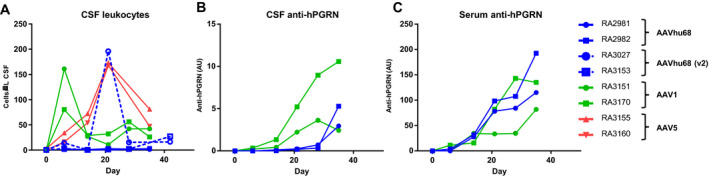
Immune response to human PGRN in vector‐treated NHPs. After administering AAV1, AAV5, or AAVhu68 vectors (3 × 10^13^ GC) expressing human PGRN from a chicken beta‐actin promoter or a ubiquitin C promoter (AAVhu68 V2) into the cisterna magna, we collected CSF on a weekly basis for chemistry and cytology analysis. A, Increased leukocyte counts (predominantly small lymphocytes) were evident in most animals. We measured antibody responses to human PGRN by ELISA in (B) CSF and (C) serum of animals treated with AAVhu68 or AAV1. We did not evaluate antibodies against human PGRN in serum or CSF samples from animals treated with AAV5.

ICM AAV delivery was well tolerated in all treatment groups. We did not identify any treatment‐related abnormalities on daily observations, physical exams, complete blood counts, or serum chemistry panels. We tested the CSF and plasma samples from NHPs treated with AAVhu68 and AAV1 vectors carrying the beta‐actin promoter for antibodies to human PGRN. All four animals developed antibodies to the human transgene product (Fig. 4). The kinetics of the anti‐human PGRN antibody response in CSF correlated with transgene expression levels, peaking earlier in the AAV1 group. Similar to other ICM AAV studies that utilize a xenogenic transgene,^25^ CSF analysis revealed an asymptomatic lymphocytic pleocytosis beginning 7–21 days after injection for all vector serotypes, mirroring the antibody response to the transgene product (Fig. 4). CSF cell counts declined from peak levels but remained elevated at the time of necropsy for most animals.

After 35 days following injection, we sacrificed the animals treated with the two highest expressing vectors—AAV1 and AAVhu68 carrying the beta‐actin promoter—to evaluate the histopathology of the brain and spinal cord. Our findings were similar to previous ICM AAV studies.^25^, ^26^ We observed occasional, minimal lymphocytic infiltrates in the meninges and choroid plexus as well as degeneration of sensory neurons and their associated axons in some dorsal root ganglia and spinal cord sections (Fig. S3). The sensory neuron findings in dorsal root ganglia and axonal degeneration of dorsal white‐matter tracts in the spinal cord were generally minimal to mild in severity for AAV1 and AAVhu68‐treated animals. These findings were consistent with previous ICM AAV studies and were not associated with clinical signs.^21^, ^25^, ^26^ No vector‐related abnormalities were noted in the brain parenchyma of any of the AAV1‐ or AAVhu68‐treated animals.

### Differing patterns of CNS transduction following ICM administration of AAV1 and AAVhu68 vectors to NHPs

The markedly higher PGRN levels in the CSF of NHPs treated with an AAV1 vector led us to explore the differences in the transduction patterns of AAV1, AAV5, and AAVhu68 in the primate CNS. We administered a single ICM injection of an AAV1, AAV5, or AAVhu68 vector (3 × 10^13^ GC) expressing a green fluorescent protein (GFP) reporter gene to adult NHPs (n = 2 per vector).

**Figure 5:**
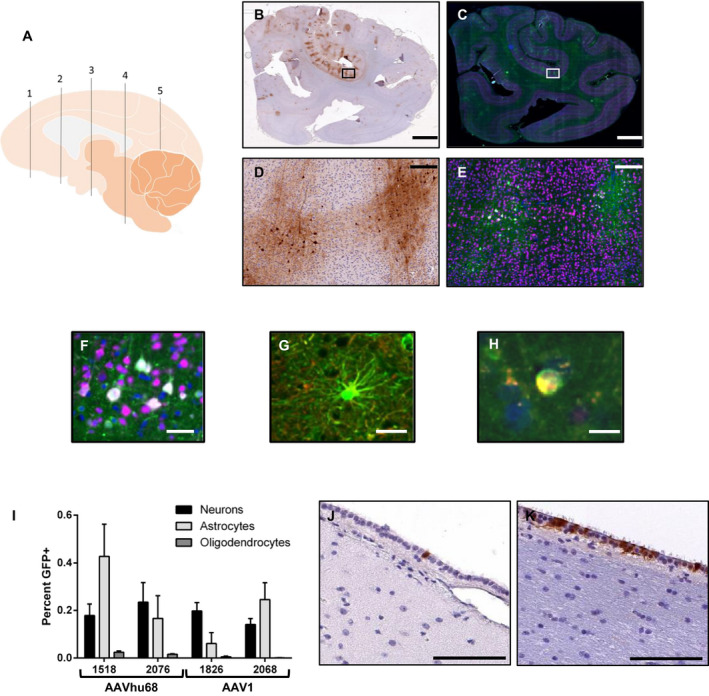
Characterization of brain transduction following ICM administration of AAV1 and AAVhu68 vectors to NHPs. Adult rhesus macaques received 3 × 10^13^ GC of AAVhu68 (N = 2) or AAV1 (N = 2) vectors expressing GFP from a chicken beta‐actin promoter by ICM injection. Animals were necropsied 28 days after vector administration. A, We analyzed sections of five regions of the right hemisphere of the brain by (B, inset D) GFP immunohistochemistry or (C, inset E) immunofluorescence with staining for GFP (green) and 4′,6‐diamidino‐2‐phenylindole (DAPI; blue). Co‐staining with markers of specific cell types (NeuN, GFAP, and Olig2) allowed us to quantify transduced (C, E, F) neurons, (G) astrocytes, and (H) oligodendrocytes. I, We calculated the mean transduction of each cell type for all sampled brain regions. We evaluated ependymal cell transduction by performing immunohistochemistry in multiple regions of the lateral ventricle and fourth ventricle of animals treated with AAVhu68 (J) and animal RA1826, who was treated with AAV1 (K). Error bars = standard error of the mean (SEM) of the five sections. Scale bars = 5 mm (B, C), 200 µm (D, E). 100 µm (J, K), 50 µm (F, G), 10 µm (H).

Twenty‐eight days after injection, immunohistochemistry revealed diffuse, patchy transduction throughout the brains of NHPs treated with AAV1 or AAVhu68 vectors (Fig. 5). Minimal transduction was evident in the brains of animals that received the AAV5 vector. In order to precisely characterize the differences in transduction between AAV1 and AAVhu68, we developed a semi‐automated method to quantify transduced cells in sections collected from multiple brain regions. Using sections stained with fluorescently labeled antibodies against GFP and markers of specific cell types, we quantified the total numbers of neurons, oligodendrocytes, microglia and astrocytes using NeuN, Olig2, IBA1, and GFAP staining, respectively, and then quantified GFP‐expressing cells of each type (Fig. 5, Table S1). AAV1 and AAVhu68 each transduced less than 1% of each cell type in all examined regions. Transduction of neurons was nearly equivalent between the two vectors. However, AAVhu68 appeared to transduce modestly greater numbers of astrocytes and oligodendrocytes. We did not observe transduction of microglia in any sections examined.

The roughly equivalent brain transduction observed with AAV1 and AAVhu68 vectors was unexpected given the dramatically higher CSF PGRN levels achieved with AAV1. Interestingly, multiple brain sections from an AAV1‐treated animal (RA1826) that contained portions of the ventricular system demonstrated extensive transduction of the ependymal cells that line the ventricles, which we did not observe in either AAVhu68‐treated animal (Fig. 5). An average of 48% of ependymal cells were transduced across all sampled regions, including the frontal, temporal, and occipital horn of the lateral ventricle as well as the fourth ventricle. In contrast, only 1‐2% of ependymal cells were transduced in the same brain regions of the animals that were given the AAVhu68 vector. We were only able to evaluate small segments of one lateral ventricle in the second AAV1‐treated animal, which showed approximately 1% ependymal cell transduction, although the analysis was limited to the small sampled region. These findings suggest that highly transduced ependymal cells in AAV1‐treated animals could be the primary source of PGRN in the CSF given that the transduction of other cells types appeared to be similar across the two serotypes.

## Discussion

ICM AAV delivery offers a potential minimally invasive approach to achieve durable and widespread PGRN delivery to the CNS of *GRN* mutation carriers.^13^, ^27^ In the absence of an authentic animal model of FTD‐GRN, we evaluated the impact of AAV‐mediated PGRN expression on lysosomal abnormalities in *GRN* ^−/−^ mice. Recent studies revealed that PGRN is a downstream target for a lysosomal master‐regulator transcription factor EB.^28^, ^29^ PGRN is also a promoter of lysosome acidification and a chaperone for lysosomal proteases.^5^, ^6^ Transcriptomic and proteomic analysis of *GRN* ^−/−^ mice showed upregulation of lysosome‐related genes,^16^, ^20^ further establishing the importance of PGRN for proper lysosomal function. These findings are consistent with the prominent lysosomal storage lesions observed in the brains of patients with NCL and FTD caused by *GRN* mutations.^3^, ^4^ Therefore, we used markers of lysosomal function as the primary readout for proof‐of‐concept studies. ICV delivery of an AAVhu68 vector expressing human PGRN reduced lysosomal lipofuscin deposits and normalized lysosomal enzymatic activity in the brains of *GRN* ^−/−^ mice. Microgliosis, a prominent finding in brains of FTD‐GRN patients, was also reversed by vector administration in the murine disease model. These findings demonstrate that *GRN* gene delivery can effectively correct key aspects of the underlying pathophysiology of *GRN*‐related neurodegenerative disease.

Recently, another group raised a potential safety concern for AAV‐mediated PGRN expression, reporting that ICV administration of an AAV9 vector expressing human PGRN in mice caused T‐cell infiltration and extensive hippocampal degeneration.^30^ Similar T‐cell responses to a xenogenic transgene product following AAV delivery to the brain have been extensively described in the literature,^27^, ^31^, ^32^, ^33^ particularly when the vector is administered into the lateral ventricle or the brain parenchyma, which can provoke a local inflammatory response. In our mouse study using AAVhu68, a variant of AAV9, we did not observe any lymphocyte infiltration or gross morphological abnormalities in the hippocampus two or four months after injection. Another group that delivered AAV1‐expressing human PGRN to the mouse brain reported only a minor, localized cellular infiltrate at the injection site two months after injection.^10^ These apparently contradictory results could stem from differences in vector production or formulation, technical aspects of injections, study duration, or differences in vector serotype. Regardless, the finding of a cytotoxic T‐cell response to a foreign transgene in a knockout mouse is likely of no relevance to gene therapy for heterozygous *GRN* mutation carriers, which express normal PGRN protein, albeit at reduced levels, and are expected to be immunologically tolerant to the protein. In addition, we previously found that vector administration via ICM injection, rather than injection into the lateral ventricle, can dramatically reduce the risk of a T‐cell response to even a highly immunogenic transgene.^27^

To assess the safety and feasibility of *GRN* gene transfer in a more clinically relevant system, we performed a study in NHPs. The size and anatomic similarity of NHPs to humans allowed us to replicate the ICM route of administration that would be employed in human trials. ICM vector injection in rhesus macaques was well tolerated. All animals eventually developed antibodies to the human PGRN protein in CSF, which was accompanied by a lymphocytic pleocytosis. As with immune responses to human PGRN in mouse models, the antibody response to the human transgene product observed in NHPs is not expected to be relevant to FTD‐GRN patients, who are immunologically tolerant to the protein due to residual endogenous expression.

Detailed quantification of transduced cells in the primate brain following ICM AAV delivery revealed that while transduction is widespread, only a small fraction of neurons, astrocytes, and oligodendrocytes are transduced. Both AAV1 and AAVhu68—a vector closely related to AAV9 with similar transduction characteristics—transduced less than 1% of neurons at a moderate vector dose. Transduction efficiency of AAV5 was even lower, precluding analysis by the same methods used for AAV1 and AAVhu68‐treated animals. Any assessment of transduction efficiency must be interpreted with caution due to potential biases introduced by the sensitivity of the method used to detect the transgene product or the brain regions sampled. However, our findings clearly suggest that the current approach of AAV delivery into CSF will be inadequate to address diseases of the CNS in which the transgene product must be expressed in a significant fraction of neurons or glia. This is a critical observation for other genetic forms of FTD, such as those caused by C9ORF72 or MAPT mutations, where gene therapy approaches will be far more challenging because of the cell‐autonomous toxicity caused by these mutations. In contrast, the bystander effect mediated by secreted PGRN makes FTD caused by *GRN* mutations uniquely amenable to AAV gene therapy.

As extracellular PGRN can be taken up by neurons, the remarkably high CSF PGRN levels achieved with the AAV1 vector—apparently mediated by robust ependymal cell transduction—could make AAV1 an ideal choice for *GRN* gene therapy. Others have reported that AAV1 transduces ependymal cells after delivery into the lateral cerebral ventricles.^34^ We found that AAV1 also appears to effectively target ependymal cells after ICM delivery. However, ependymal cell transduction could only be adequately assessed in one animal, and future studies should further evaluate this finding. The ependymal cells of the brain are believed to be post‐mitotic and may be an effective depot for long‐term production of therapeutic proteins in the CSF.^35^, ^36^ Although the minimum therapeutic concentration of PGRN in CSF is unknown, our studies in *GRN* ^−/−^ mice demonstrate that expression of PGRN in CSF at tenfold normal human levels is sufficient to normalize histological and biochemical markers of *GRN* deficiency throughout the brain. NHPs treated with an AAV1 vector expressed up to 40‐fold normal human PGRN levels in CSF, indicating that therapeutic expression levels of PGRN may be achievable with ICM AAV1 delivery.

## Author Contributions

C.H., R.M., C.D., J.J., P.B., and E.B. designed and performed experiments. J.M.W. designed experiments. C.H., R.M., and J.M.W. wrote the manuscript.

## Conflict of Interest

J.M. Wilson is a paid advisor to and holds equity in Scout Bio and Passage Bio; he holds equity in Surmount Bio; he also has a sponsored research agreement with Ultragenyx, Biogen, Janssen, Precision Biosciences, Moderna Inc., Scout Bio, Passage Bio, Amicus Therapeutics, and Surmount Bio which are licensees of Penn technology. JMW is an inventor on patents that have been licensed to various biopharmaceutical companies and for which he may receive payments. C. Hinderer is an inventor on patents licensed to biopharmaceutical companies and holds equity in Scout Bio.

## Supporting information


**Supplemental Figure S1.** Natural history of lipofuscin accumulation and hexosaminidase activity in brains of GRN^−/−^ mice. GRN^−/−^ mice (KO) or GRN^+/+^ (WT) controls were sacrificed at the ages indicated (n = 10 per time point). Unstained brain sections were imaged for autofluorescent material (lipofuscin) in hippocampus, thalamus and frontal cortex, and lipofuscin deposits were quantified by three blinded reviewers and averaged (A‐C). Lipofuscin counts are expressed relative to the total area of the region of interest. Hexosaminidase activity was measured in brain samples and normalized to total protein concentration (D). Values are expressed as a ratio to wild‐type controls.

**Supplemental Figure S2.** AAV‐mediated expression of human PGRN corrects lysosomal pathology in brains of aged GRN^−/−^ mice. GRN^−/−^ mice (KO) or GRN^+/+^ (WT) controls were treated with a single ICV injection of vehicle (PBS) or an AAVhu68 vector expressing human PGRN (10^11^ GC) at 7 months of age. Animals were sacrificed 4 months after injection. Hexosaminidase activity was measured in brain samples (A) and lipofuscin deposits were quantified in hippocampus, thalamus and cortex by a blinded reviewer (B‐D). Lipofuscin counts are expressed per high power field. *P < 0.05, **P < 0.005, one‐way ANOVA followed by Tukey’s multiple comparisons test.

**Supplemental Figure S3.** Sensory neuron lesions in NHPs treated with AAV1 or AAVhu68 expressing human PGRN. Adult rhesus macaques were administered 3 x 10^13^ GC AAVhu68 (n = 2) or AAV1 (n = 2) vectors expressing human PGRN from a chicken beta actin promoter by ICM injection on study day 0. Animals were necropsied 35 days after vector administration. H&E stained sections of DRGs and spinal cord from the cervical, thoracic and lumbar levels were examined in a blinded manner by a board‐certified veterinary pathologist. Findings of neuronal degeneration (DRG) and axonal degeneration (spinal cord dorsal columns) were assigned a score of 0 (absent), 1 (minimal), 2 (mild), 3 (moderate), 4 (marked) or 5 (severe). The total score was calculated by adding the scores from the cervical, thoracic and lumbar sections. The maximum severity score is 15.

**Supplemental Table S1.** Percent neuron, astrocyte, and oligodendrocyte transduction following ICM administration of AAV1 and AAVhu68 vectors to nonhuman primates. Adult rhesus macaques were administered 3 x 10^13^ GC AAVhu68 (n = 2) or AAV1 (n = 2) vectors expressing GFP from a chicken beta actin promoter by ICM injection on study day 0. Animals were necropsied 28 days after vector administration, and sections of five regions (shown in figure 5) of the right hemisphere of the brain were analyzed by GFP immunofluorescence with costaining for specific cell types (NeuN, GFAP and Olig2). Total cells of each cell type and the number of GFP expressing cells of each type were quantified using HALO software. The percentage of each cell type transduced is shown for each region. For some animals, two sections were analyzed from region 5.

**Supplementary Material and Methods.** Vector production, Histology and imaging, Sample preparation for the hexosaminidase (Hex) assay, ELISA, Neutralizing antibody assay.

## References

[1] Gass J , Cannon A , Mackenzie IR , et al. Mutations in progranulin are a major cause of ubiquitin‐positive frontotemporal lobar degeneration. Hum Mol Genet 2006 15(20):2988–3001. 16950801 10.1093/hmg/ddl241

[2] Caswell C , McMillan CT , Xie SX , et al. Genetic predictors of survival in behavioral variant frontotemporal degeneration. Neurology 2019;93(18):e1707–e1714. 31537715 10.1212/WNL.0000000000008387 PMC6946477

[3] Smith Katherine R , Damiano J , Franceschetti S , et al. Strikingly different clinicopathological phenotypes determined by progranulin‐mutation dosage. Am J Hum Genet 2012, 90(6):1102–1107. 22608501 10.1016/j.ajhg.2012.04.021 PMC3370276

[4] Gotzl JK , Mori K , Damme M , et al. Common pathobiochemical hallmarks of progranulin‐associated frontotemporal lobar degeneration and neuronal ceroid lipofuscinosis. Acta Neuropathol 2014;127(6):845–860. 24619111 10.1007/s00401-014-1262-6

[5] Beel S , Moisse M , Damme M , et al. Progranulin functions as a cathepsin D chaperone to stimulate axonal outgrowth in vivo. Hum Mol Genet 2017;26(15):2850–2863. 28453791 10.1093/hmg/ddx162 PMC5886064

[6] Tanaka Y , Suzuki G , Matsuwaki T , et al. Progranulin regulates lysosomal function and biogenesis through acidification of lysosomes. Hum Mol Genet 2017;26(5):969–988. 28073925 10.1093/hmg/ddx011

[7] Siintola E , Partanen S , Stromme P , et al. Cathepsin D deficiency underlies congenital human neuronal ceroid‐lipofuscinosis. Brain 2006;129(Pt 6):1438–1445. 16670177 10.1093/brain/awl107

[8] Zhou X , Sun L , Bastos de Oliveira F , et al. Prosaposin facilitates sortilin‐independent lysosomal trafficking of progranulin. J Cell Biol 2015;210(6):991–1002. 26370502 10.1083/jcb.201502029 PMC4576858

[9] Arrant AE , Filiano AJ , Unger DE , et al. Restoring neuronal progranulin reverses deficits in a mouse model of frontotemporal dementia. Brain 2017;140(5):1447–1465. 28379303 10.1093/brain/awx060 PMC5965303

[10] Arrant AE , Onyilo VC , Unger DE , Roberson ED . Progranulin gene therapy improves lysosomal dysfunction and microglial pathology associated with frontotemporal dementia and neuronal ceroid lipofuscinosis. J Neurosci 2018;38(9):2341–2358. 29378861 10.1523/JNEUROSCI.3081-17.2018 PMC5830520

[11] Gray SJ , Nagabhushan Kalburgi S , McCown TJ , Jude SR . Global CNS gene delivery and evasion of anti‐AAV‐neutralizing antibodies by intrathecal AAV administration in non‐human primates. Gene Ther 2013;20(4):450–459. 23303281 10.1038/gt.2012.101 PMC3618620

[12] Samaranch L , Salegio EA , San Sebastian W , et al. Strong cortical and spinal cord transduction after AAV7 and AAV9 delivery into the cerebrospinal fluid of nonhuman primates. Hum Gene Ther 2013;24(5):526–532. 23517473 10.1089/hum.2013.005 PMC3655626

[13] Hinderer C , Bell P , Vite CH , et al. Widespread gene transfer in the central nervous system of cynomolgus macaques following delivery of AAV9 into the cisterna magna. Mol Ther Methods Clin Dev 2014;1:14051. 26052519 10.1038/mtm.2014.51 PMC4448732

[14] Katz N , Goode T , Hinderer C , et al. Standardized method for intra‐cisterna magna delivery under fluoroscopic guidance in nonhuman primates. Hum Gene Ther Method 2018;29(5):212–219. 10.1089/hgtb.2018.041 30032644

[15] Karageorgos LE , Isaac EL , Brooks DA , et al. Lysosomal Biogenesis in Lysosomal Storage Disorders. Exp Cell Res 1997;234(1):85–97. 9223373 10.1006/excr.1997.3581

[16] Lui H , Zhang J , Makinson SR , et al. Progranulin deficiency promotes circuit‐specific synaptic pruning by microglia via complement activation. Cell 2016;165(4):921–935. 27114033 10.1016/j.cell.2016.04.001 PMC4860138

[17] Ahmed Z , Sheng H , Xu YF , et al. Accelerated lipofuscinosis and ubiquitination in granulin knockout mice suggest a role for progranulin in successful aging. Am J Pathol 2010;177(1):311–324. 20522652 10.2353/ajpath.2010.090915 PMC2893674

[18] Arrant AE , Filiano AJ , Warmus BA , et al. Progranulin haploinsufficiency causes biphasic social dominance abnormalities in the tube test. Genes Brain Behav 2016;15(6):588–603. 27213486 10.1111/gbb.12300 PMC5943713

[19] Ward ME , Chen R , Huang HY , et al. Individuals with progranulin haploinsufficiency exhibit features of neuronal ceroid lipofuscinosis. Sci Transl Med 2017;9(385). 10.1126/scitranslmed.aah5642 PMC5526610 28404863

[20] Klein ZA , Takahashi H , Ma M , et al. Loss of TMEM106B ameliorates lysosomal and frontotemporal dementia‐related phenotypes in progranulin‐deficient mice. Neuron 2017;95(2):281–96.e6. 28728022 10.1016/j.neuron.2017.06.026 PMC5558861

[21] Gurda BL , De Guilhem De Lataillade A , Bell P , et al. Evaluation of AAV‐mediated gene therapy for central nervous system disease in canine mucopolysaccharidosis VII. Mol Ther 2016;24(2):206–216. 26447927 10.1038/mt.2015.189 PMC4817811

[22] Hinderer C , Bell P , Gurda BL , et al. Intrathecal gene therapy corrects CNS pathology in a feline model of mucopolysaccharidosis I. Mol Ther 2014;22(12):2018–2027. 25027660 10.1038/mt.2014.135 PMC4429692

[23] Hudry E , Andres‐Mateos E , Lerner EP , et al. Efficient gene transfer to the central nervous system by single‐stranded Anc80L65. Mol Ther 2018;21(10):197–209. 10.1016/j.omtm.2018.07.006 PMC6083902 30109242

[24] Ahmed Z , Mackenzie IR , Hutton ML , Dickson DW . Progranulin in frontotemporal lobar degeneration and neuroinflammation. J Neuroinflammation 2007;11(4):7. 10.1186/1742-2094-4-7 PMC1805428 17291356

[25] Hordeaux J , Hinderer C , Goode T , et al. Toxicology study of intra‐cisterna magna adeno‐associated virus 9 expressing human alpha‐L‐iduronidase in rhesus macaques. Mol Ther 2018;21(10):79–88. 10.1016/j.omtm.2018.06.003 PMC6070681 30073179

[26] Hordeaux J , Hinderer C , Goode T , et al. Toxicology study of intra‐cisterna magna adeno‐associated virus 9 expressing iduronate‐2‐sulfatase in Rhesus macaques. Molecular Ther 2018;21(10):68–78. 10.1016/j.omtm.2018.06.004 PMC6070702 30073178

[27] Hinderer C , Bell P , Katz N , et al. Evaluation of intrathecal routes of administration for adeno‐associated viral vectors in large animals. Hum Gene Ther 2018;29(1):15–24. 28806897 10.1089/hum.2017.026 PMC5770082

[28] Sardiello M , Palmieri M , di Ronza A , et al. A gene network regulating lysosomal biogenesis and function. Science 2009;325(5939):473–477. 19556463 10.1126/science.1174447

[29] Settembre C , Di Malta C , Polito VA , et al. TFEB links autophagy to lysosomal biogenesis. Science 2011;332(6036):1429–1433. 21617040 10.1126/science.1204592 PMC3638014

[30] Amado DA , Rieders JM , Diatta F , et al. AAV‐mediated progranulin delivery to a mouse model of progranulin deficiency causes T cell‐mediated toxicity. Mol Ther 2019;27(2):465–478. 30559071 10.1016/j.ymthe.2018.11.013 PMC6369714

[31] Ellinwood NM , Ausseil J , Desmaris N , et al. Safe, efficient, and reproducible gene therapy of the brain in the dog models of Sanfilippo and Hurler syndromes. Mol Ther 2011;19(2):251–259. 21139569 10.1038/mt.2010.265 PMC3034858

[32] Samaranch L , Sebastian WS , Kells AP , et al. AAV9‐mediated expression of a non‐self protein in nonhuman primate central nervous system triggers widespread neuroinflammation driven by antigen‐presenting cell transduction. Mol Ther 2014;22(2):329–337. 24419081 10.1038/mt.2013.266 PMC3918916

[33] Ciesielska A , Hadaczek P , Mittermeyer G , et al. Cerebral infusion of AAV9 vector‐encoding non‐self proteins can elicit cell‐mediated immune responses. Mol Ther 2013;21(1):158–166. 22929660 10.1038/mt.2012.167 PMC3538301

[34] Yamazaki Y , Hirai Y , Miyake K , Shimada T . Targeted gene transfer into ependymal cells through intraventricular injection of AAV1 vector and long‐term enzyme replacement via the CSF. Sci Rep 2014;07/01/online;4:5506. 10.1038/srep05506 PMC4076682 24981028

[35] Spassky N , Merkle FT , Flames N , et al. Adult ependymal cells are postmitotic and are derived from radial glial cells during embryogenesis. J Neurosci 2005;25(1):10–18. 15634762 10.1523/JNEUROSCI.1108-04.2005 PMC6725217

[36] Dindot S , Piccolo P , Grove N , et al. Intrathecal injection of helper‐dependent adenoviral vectors results in long‐term transgene expression in neuroependymal cells and neurons. Hum Gene Ther 2011;22(6):745–751. 21175294 10.1089/hum.2010.147 PMC3155126

