# Supplementary figures and images for: Adeno‐associated virus serotype 1‐based gene therapy for FTD caused by GRN mutations
Source: Ann Clin Transl Neurol. 2020 Sep 16;7(10):1843–53. doi: 10.1002/acn3.51165 (PMC7545603; doi:10.1002/acn3.51165)

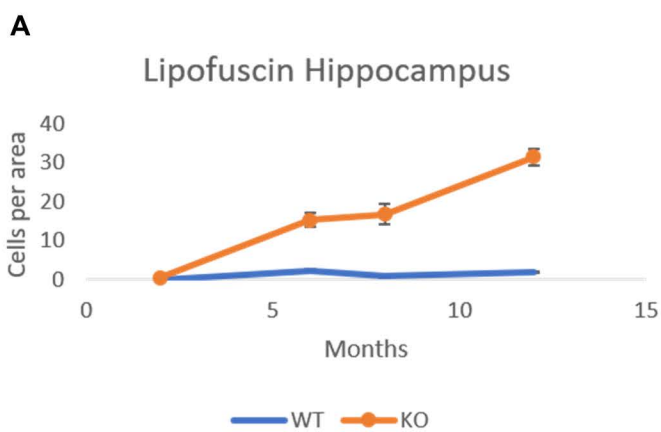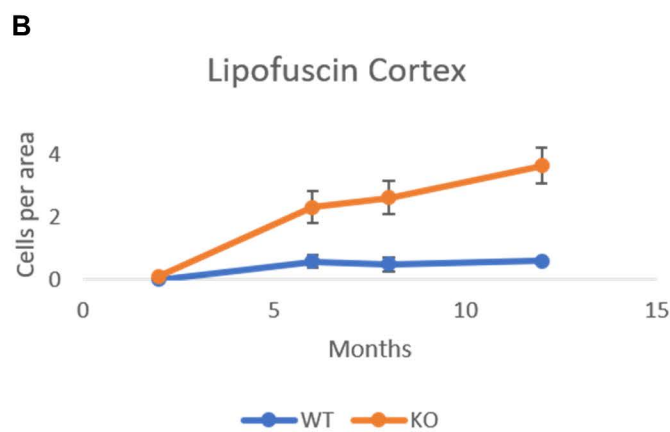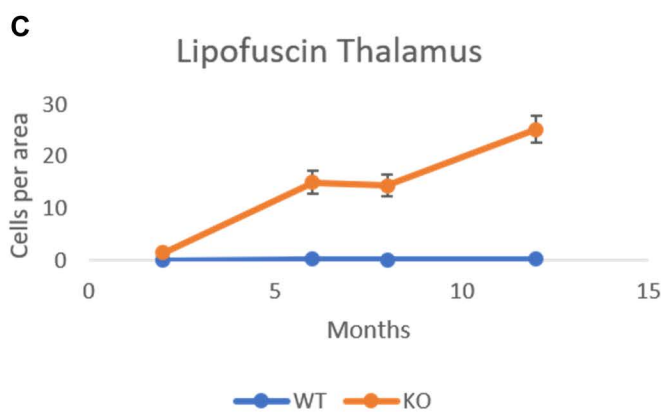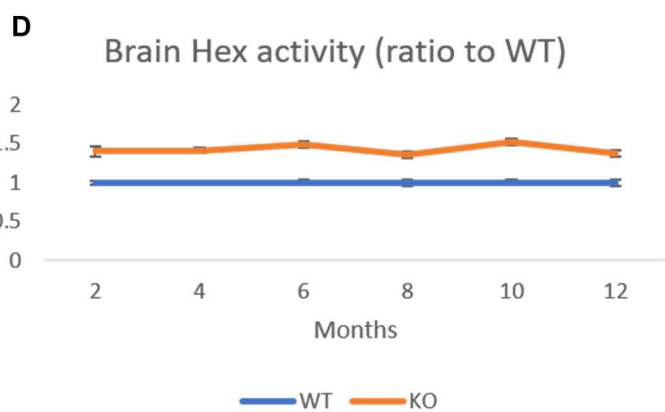

Supplement: Supplementary file 1 — Supplemental Figure S1. Natural history of lipofuscin accumulation and hexosaminidase activity in brains of GRN−/− mice. GRN−/− mice (KO) or GRN+/+ (WT) controls were sacrificed at the ages indicated (n = 10 per time point). Unstained brain sections were imaged for autofluorescent material (lipofuscin) in hippocampus, thalamus and frontal cortex, and lipofuscin deposits were quantified by three blinded reviewers and averaged (A‐C). Lipofuscin counts are expressed relative to the total area of the region of interest. Hexosaminidase activity was measured in brain samples and normalized to total protein concentration (D). Values are expressed as a ratio to wild‐type controls. [file ACN3-7-1843-s001.pdf]

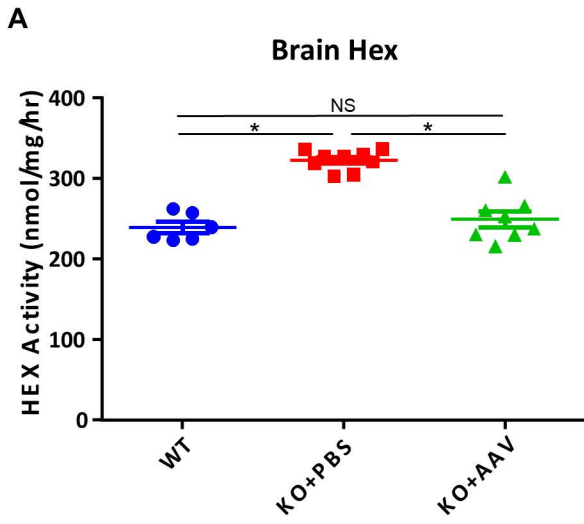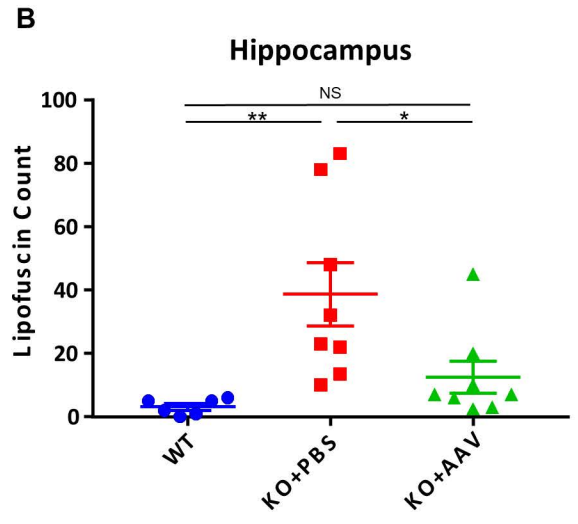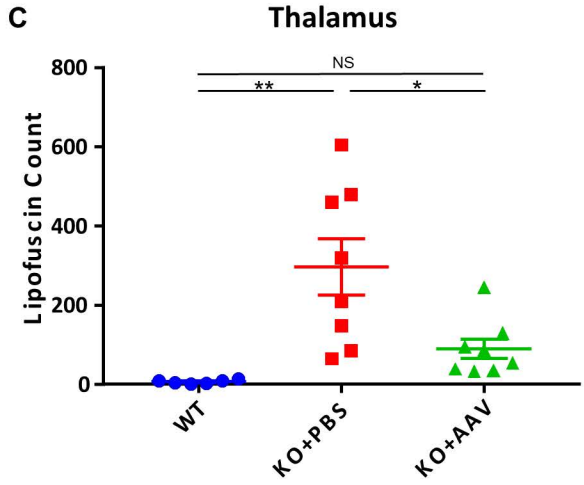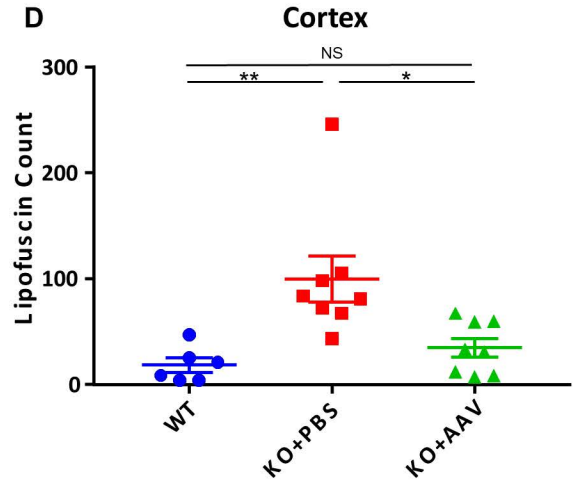

Supplement: Supplementary file 2 — Supplemental Figure S2. AAV‐mediated expression of human PGRN corrects lysosomal pathology in brains of aged GRN−/− mice. GRN−/− mice (KO) or GRN+/+ (WT) controls were treated with a single ICV injection of vehicle (PBS) or an AAVhu68 vector expressing human PGRN (1011 GC) at 7 months of age. Animals were sacrificed 4 months after injection. Hexosaminidase activity was measured in brain samples (A) and lipofuscin deposits were quantified in hippocampus, thalamus and cortex by a blinded reviewer (B‐D). Lipofuscin counts are expressed per high power field. *P < 0.05, **P < 0.005, one‐way ANOVA followed by Tukey’s multiple comparisons test. [file ACN3-7-1843-s002.pdf]

**DRG cumulative score**

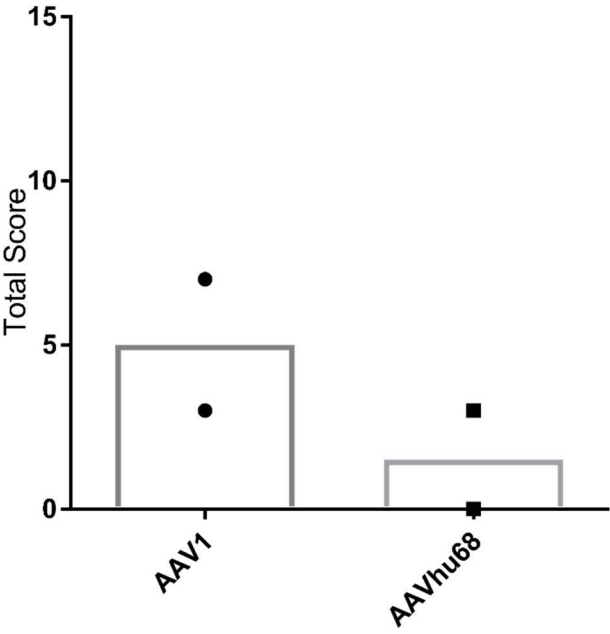

**Dorsal axonopathy cumulative score**

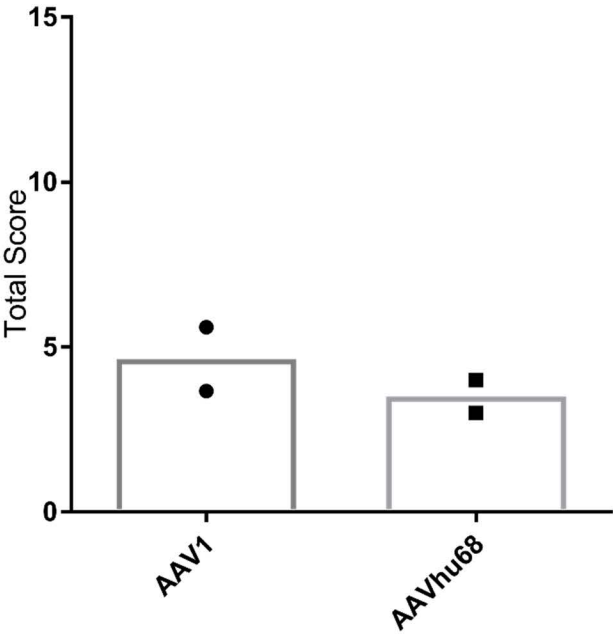

Supplement: Supplementary file 3 — Supplemental Figure S3. Sensory neuron lesions in NHPs treated with AAV1 or AAVhu68 expressing human PGRN. Adult rhesus macaques were administered 3 x 10^13^ GC AAVhu68 (n = 2) or AAV1 (n = 2) vectors expressing human PGRN from a chicken beta actin promoter by ICM injection on study day 0. Animals were necropsied 35 days after vector administration. H&E stained sections of DRGs and spinal cord from the cervical, thoracic and lumbar levels were examined in a blinded manner by a board‐certified veterinary pathologist. Findings of neuronal degeneration (DRG) and axonal degeneration (spinal cord dorsal columns) were assigned a score of 0 (absent), 1 (minimal), 2 (mild), 3 (moderate), 4 (marked) or 5 (severe). The total score was calculated by adding the scores from the cervical, thoracic and lumbar sections. The maximum severity score is 15. [file ACN3-7-1843-s003.pdf]
