# Supplementary material for: Adeno‐associated virus serotype 1‐based gene therapy for FTD caused by GRN mutations
Source: Ann Clin Transl Neurol. 2020 Sep 16;7(10):1843–53. doi: 10.1002/acn3.51165 (PMC7545603; doi:10.1002/acn3.51165)
Supplement: Supplementary file 4 — Supplemental Table S1. Percent neuron, astrocyte, and oligodendrocyte transduction following ICM administration of AAV1 and AAVhu68 vectors to nonhuman primates. Adult rhesus macaques were administered 3 x 10^13^ GC AAVhu68 (n = 2) or AAV1 (n = 2) vectors expressing GFP from a chicken beta actin promoter by ICM injection on study day 0. Animals were necropsied 28 days after vector administration, and sections of five regions (shown in figure 5) of the right hemisphere of the brain were analyzed by GFP immunofluorescence with costaining for specific cell types (NeuN, GFAP and Olig2). Total cells of each cell type and the number of GFP expressing cells of each type were quantified using HALO software. The percentage of each cell type transduced is shown for each region. For some animals, two sections were analyzed from region 5. [file ACN3-7-1843-s004.pdf]

		Region						
Cell marker	Animal ID	**1**	**2**	**3**	**4**	**5**		**Average**
NeuN	**1518**	0.13	N/A	0.205	0.34	0.167	0.049	0.178
	**2076**	0.106	0.227	0.258	0.528	0.053	N/A	0.234
	**1826**	0.1	0.318	0.195	0.202	0.171	N/A	0.197
	**2068**	0.218	N/A	0.153	0.141	0.128	0.065	0.141
GFAP	**1518**	0.152	N/A	0.202	0.914	0.39	0.476	0.427
	**2076**	0.011	0.064	0.235	0.511	0.011	N/A	0.166
	**1826**	0.019	0.013	0.021	0.244	0.007	N/A	0.061
	**2068**	0.066	N/A	0.245	0.13	0.459	0.33	0.246
Olig2	**1518**	0.02	N/A	0.03	0.04	0.009	0.023	0.024
	**2076**	0.02	0.023	0.011	0.013	0.013	N/A	0.016
	**1826**	0	0.001	0.008	0.016	N/A	0.001	0.005
	**2068**	0.002	0	N/A	0.001	0.002	0.002	0.001
