# Supplementary material for: Adeno‐associated virus serotype 1‐based gene therapy for FTD caused by GRN mutations
Source: Ann Clin Transl Neurol. 2020 Sep 16;7(10):1843–53. doi: 10.1002/acn3.51165 (PMC7545603; doi:10.1002/acn3.51165)
Supplement: Supplementary file 5 — Supplementary Material and Methods. Vector production, Histology and imaging, Sample preparation for the hexosaminidase (Hex) assay, ELISA, Neutralizing antibody assay. [file ACN3-7-1843-s005.docx]

**Supplemental Materials and Methods:**

*Vector production*

We cloned codon-optimized human *GRN* and GFP cDNAs into an expression construct containing a chicken beta-actin promotor with a cytomegalovirus early enhancer, chimeric intron, and rabbit beta-globin polyadenylation sequence. We then cloned a second codon-optimized human *GRN* cDNA into an expression construct containing the human ubiquitin C promoter. The expression constructs were flanked by AAV2 inverted terminal repeats. We generated AAV serotypes 1, 5, and AAVhu68 from these constructs via the triple transfection of HEK293 cells and iodixanol purification as previously described [^1^](#_ENREF_1).

*Histology and imaging*

After fixing mouse brains in 10% formalin, we cryo-preserved the tissue in sucrose and embedded the tissue in an optimal cutting-temperature compound. We then cryostat sectioned the tissue. We captured low-magnification images of auto-fluorescent material (lipofuscin) within the regions of interest. A blinded reviewer quantified lipofuscin deposits using Image J software. We fixed NHP tissues in 10% formalin, which we then paraffin embedded and stained with hematoxylin and eosin. A board-certified veterinary anatomic pathologist reviewed the slides. For animals treated with GFP vectors, we stained the brain sections with antibodies against Olig2, GFAP, or NeuN. We co-stained all sections with DAPI and an antibody against GFP, followed by fluorescent secondary antibodies. We scanned the slides on a Leica Aperio Versa 200 slide scanner. We then downloaded the slides from eSlide Manager to analyze them using HALO imaging software (Indica Labs). For each animal, we sampled five regions of the right hemisphere; cells with each cell-type marker were quantified. We detected cells by adjusting the following settings: “minimum nuclear intensity”, “nuclear size”, “nuclear segmentation aggressiveness”, and “minimum nuclear roundness” under the nuclei-detection tab. Then, we defined the criteria for each individual dye to further identify cells and generate a total cell count for each marker. We empirically determined the settings based on the sensitivity and reliability of detection of the desired cell type. In some cases, settings such as NeuN detection in the cytoplasm did not reflect the true intracellular localization of the marker, yet provided greater specificity and sensitivity. We manually verified all cells detected by automated means. For neurons, under the “dye 1” tab, we adjusted the “nucleus-positive threshold” and “cytoplasm-positive threshold” to detect only cells that had NeuN in both the nucleus and cytoplasm. For astrocytes, we selected DAPI and GFAP markers, both of which had to be present in a cell’s nucleus and cytoplasm in order to be included in the count. For oligodendrocytes, we counted a cell if DAPI and Olig2 were both present in the nucleus, but not the cytoplasm. We used the same settings for co-localization. However, we included GFP as an additional dye in the nucleus for neurons, and in both the nucleus and cytoplasm for astrocytes. Cells that did not express all selected markers were eliminated from the generated results table by using the nucleus or cytoplasm “mask” function. Because of the scarcity of GFP-positive cells co-localized with Olig2, we manually counted transduced oligodendrocytes. In some cases, blood vessels or portions of the choroid plexus exhibited autofluorescence and were manually outlined and excluded using the “scissors” tool. The resulting values were expressed as percentages of GFP-positive cells for each cell type marker.

*Sample preparation for the hexosaminidase (Hex) assay*

We directly used serum in the Hex-activity assay while brain samples were homogenized in lysis buffer (0.2 % Triton-X100, 0.9% NaCl, pH 4.0) followed by three freeze-thaw cycles and clarification by centrifugation. We determined protein concentrations using the Bradford assay. We performed Hex activity measurements as previously described [^2^](#_ENREF_2).

*ELISA*

We measured human PGRN using a DuoSet ELISA kit (R&D # DY2420) with minor modifications. Briefly, we coated high-binding polystyrene ELISA plates overnight at 4**°**C with 5 µg/ml human PGRN capture antibody diluted in PBS. After washing, we blocked the plates with 1% bovine serum albumin in PBS for two hours, followed by sample incubation for one hour. Human and NHP CSF was diluted 1:5, and murine CSF samples were diluted 1:40 in PBS. We diluted the brain samples to 2 mg/ml total-protein concentration in lysis buffer. We detected bound antibody with biotinylated mouse anti-human PGRN antibody and streptavidin-horseradish peroxidase. We developed plates using tetramethylbenzidine substrate for 20 minutes and the reaction was stopped with 2 N sulfuric acid before measuring absorbance at 450 nm.

*Neutralizing antibody assay*

We evaluated neutralizing antibodies against AAVhu68 as previously described [^3^](#_ENREF_3).

**Supplemental References:**

1. Lock M, Alvira M, Vandenberghe LH, et al. Rapid, simple, and versatile manufacturing of recombinant adeno-associated viral vectors at scale. *Hum Gene Ther.* Oct 2010;21(10):1259-1271.

2. Hinderer C, Bell P, Gurda BL, et al. Intrathecal gene therapy corrects CNS pathology in a feline model of mucopolysaccharidosis I. *Molecular therapy : the journal of the American Society of Gene Therapy.* Dec 2014;22(12):2018-2027.

3. Calcedo R, Vandenberghe LH, Gao G, Lin J, Wilson JM. Worldwide epidemiology of neutralizing antibodies to adeno-associated viruses. *J Infect Dis.* Feb 1 2009;199(3):381-390.
